# Evaluation of the effects of asymmetric lumbosacral transitional vertebra on pelvic morphology in dogs using ventrodorsal radiographs

**DOI:** 10.1186/s13028-024-00785-3

**Published:** 2025-01-13

**Authors:** Jon Andre Berg, Bente Kristin Saevik, Frode Lingaas, Catrine Trangerud

**Affiliations:** 1 Department of Preclinical Sciences and Pathology, Faculty of Veterinary Medicine, Norwegian University of Life Sciences, Oluf Thesens Vei 30, Ås, 1432 Norway; 2 AniCura Jeløy Dyresykehus, Varnaveien 43d, Moss, 1526 Norway; 3 Department of Companion Animal Clinical Sciences, Faculty of Veterinary Medicine, Norwegian University of Life Sciences, Oluf Thesens Vei 30, Ås, Norway; 4 Veterinaerradiologene AS, Skytta terrasse 2, Hagan, 1481 Norway

**Keywords:** Canine, Diagnostic imaging, Hip dysplasia, LTV

## Abstract

**Background:**

A lumbosacral transitional vertebra (LTV) is a congenital anomaly of the caudal vertebral column. It has been associated with asymmetrical canine hip dysplasia (CHD) and cauda equina syndrome (CES) in German Shepherd dogs. This retrospective cross-sectional study aims to report the potential influence of asymmetric LTV on pelvic anatomy using ventrodorsal (VD) radiographs.

**Results:**

The results are based on the evaluation of VD radiographs of 13,950 dogs from 14 breeds; an LTV was identified in 18.5%. The LTV segments were allotted into symmetrical (78.6%) and asymmetrical (21.4%) categories. An asymmetrical CHD grade was observed in 12.4% of the dogs, of which 39.7% had asymmetrical LTV. An asymmetric LTV was associated with an uneven sacroiliac joint length, in which the shortest sacroiliac joint is positioned more caudally, resulting in a reduced distance to the hip joint (*P* < 0.001). Rotation of the asymmetrical LTV segment about the long axis was associated with opposite pelvis rotation vertically (*P* < 0.001). Also, long-axis rotation of the asymmetric LTV segment was associated with an elevation of the pelvis (*P* < 0.001), promoting an asymmetrical CHD grade (*P* < 0.001).

**Conclusions:**

This study suggests a compensatory mechanism for the sacroiliac joint related to an asymmetrical LTV. Counter-rotation between the pelvis and the LTV segment vertically may straighten the lower back. The asymmetrical LTV segment most likely affects the rotation of the pelvis and may indirectly promote an asymmetrical CHD grade.

## Background

A lumbosacral transitional vertebra (LTV) is a congenital anomalously formed vertebra that can morphologically resemble both the lumbar and sacral segments of the spine [1–5].

Several classification systems are available for LTV in dogs [3, 4, 6]. An LTV can be classified into three types based on morphology evaluated on ventrodorsal (VD) radiography [6]. Regardless of the classification system used for LTV, the different types are usually grouped into symmetrical or asymmetrical morphology [3, 4, 7, 8].

It has been recognised that LTV, predominantly the asymmetrical morphology, has the potential to be clinically important. It has been reported that asymmetrical LTV is related to rotational alterations of the pelvis, which might promote the development of asymmetrical canine hip dysplasia (CHD) grades [7, 8]. In a recent study the authors identified an association between both symmetrical and asymmetrical LTV and hip dysplasia in dogs [9]. Furthermore, LTV is reported to predispose to cauda equina syndrome (CES) in German shepherd dogs [10, 11]. The CES is believed to result from altered local biomechanics, for which one theory is counter-rotation of the sacrum in relationship to the LTV segment [3]. In the presence of an asymmetrical LTV, the left and right sacroiliac contact areas usually differ, of which the shorter side has been deemed weaker than the larger contralateral contact area [7, 12]. This theory is not proven and not consistently reported [13]. The side with the reduced contact area is also reported to be positioned further caudally on the ilium [3, 12].

This paper builds on previous work on counter-rotation between the LTV, sacrum and pelvis and the observation of a unilateral caudally positioned sacroiliac joint with a reduced contact area [3, 7, 12]. We hypothesised that asymmetrical LTV would prompt compensatory rotational adjustments in the body to align the head-tail line. Our study aimed to explore the impact of asymmetrical LTV on pelvic morphology evaluated radiographically. Specifically, whether pelvic rotation in the vertical axis opposes the rotational direction of the asymmetric LTV segment in both the vertical and long axes. Furthermore, we aimed to investigate the proposed theory concerning asymmetrical LTV and its impact on the sacroiliac joint. We sought to determine if the reduced contact area was positioned further caudally on the pelvis and to examine whether this reduction decreased the distance between the affected sacroiliac joint and the ipsilateral hip joint. This potential reduction in distance could offer insights into previous findings, suggesting no association between overload of the reduced contact area of the sacroiliac joint [13]. Finally, we explored the association between asymmetrical LTV and an asymmetrical CHD grade.

## Methods

### Study sample

**Fig. 1:**
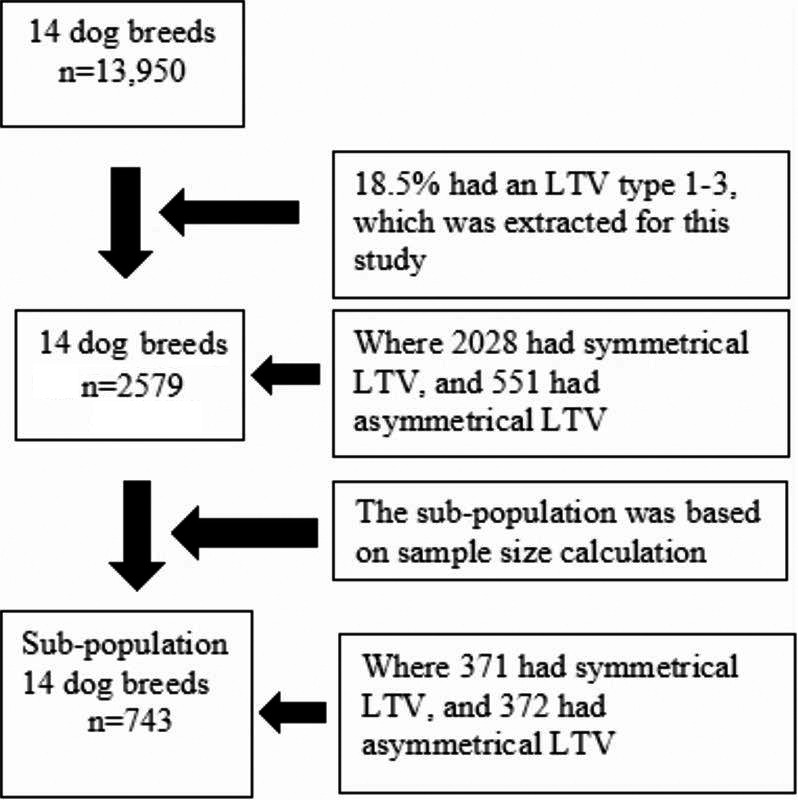
Flowchart summarising dog selection and lumbosacral transitional vertebra (LTV) classification across 14 breeds. The flowchart details the selection process and classification of lumbosacral transitional vertebra (LTV) types 1–3 from 13,950 dogs initially screened across 14 breeds [9]. Out of this initial group, 18.5% (*n* = 2,579) had LTV type 1–3, with 2,028 exhibiting symmetrical LTV and 551 showing asymmetrical LTV. A sub-population was further selected based on sample size calculation criteria, resulting in 743 dogs. Within this sub-population, 371 dogs had symmetrical LTV and 372 had asymmetrical LTV

The study sample used in this study is described in a prior publication [9]. This study was based on 13,950 dogs’ routine VD radiographs from 14 breeds stored in the Norwegian Kennel Club (NKK) database as part of the official radiographic screening program for CHD in Norway (Fig. 1). Digital radiographs were collected from February 2014 to January 2022 for a thorough classification of LTVs. The 14 dog breeds were of different sizes and groups (Table 1) [9].

**Table 1:** Information on the number of dogs within each breed

Dog breeds	Counts	% of total
Brittany	343	13.4
Bernese Mountain Dog	93	3.6
Boxer	148	5.7
Rough Collie	189	7.3
Danish-Swedish Farm Dog	149	5.8
English Setter	178	6.9
Eurasier	174	6.7
Gordon Setter	113	4.4
Irish Setter	137	5.3
Norwegian Elkhound Grey	240	9.3
Portuguese Water Dog	146	5.7
Rhodesian Ridgeback	223	8.6
German Shepherd Dog	243	9.4
Norwegian Elkhound Black	203	7.9

The inclusion criteria were as follows: an official identity number and complete records, including date of birth, sex, radiology date, and official bilateral radiological CHD grade. Due to the official criteria for the CHD grading, all dogs included were at least 12 months old at the time of radiography.

### Classification of LTV and CHD

We classified LTV into three types [6]: LTV type 1 is characterised by an independent spinous process of the first sacral vertebra, which is separated from the median sacral crest; LTV type 2 is a symmetrical transitional vertebra separated from the sacrum by an intervertebral disc space; and LTV type 3 is characterised by asymmetrical morphology. Radiographs were excluded if relevant anatomy was obscured by the os penis or dense rectal faecal material or if there was no reference point for a normal last lumbar vertebra. Two authors (the first and last author) individually evaluated the radiographs and assessed the morphology of the lumbosacral spine, reaching a consensus on cases that diverged [9]. No differentiation between lumbarisation and sacralisation was feasible based on the materials available.

The bilateral official hip joint status was based on the Federation Cynologique Internationale (FCI) classification. The FCI classification is a five-grade system from A (reflecting a normal hip joint), B (near normal hip joints), A and B are considered as free of hip dysplasia, C (mild hip dysplasia), D (moderate hip dysplasia), and E (indicating severe hip dysplasia). Grades are defined descriptively based on the Norberg angle, subluxation degree, acetabulum’s shape and depth and secondary signs of osteoarthritis [14].

### Rotational changes of the LTV, sacrum and pelvis

For consistency with previous publications using VD radiographs for evaluation of CHD to evaluate the rotational alteration of the LTV, sacrum and pelvis, the terms vertical and long axis are used. Rotational changes were classified as no rotation, left or right rotation [3, 8].

**Fig. 2:**
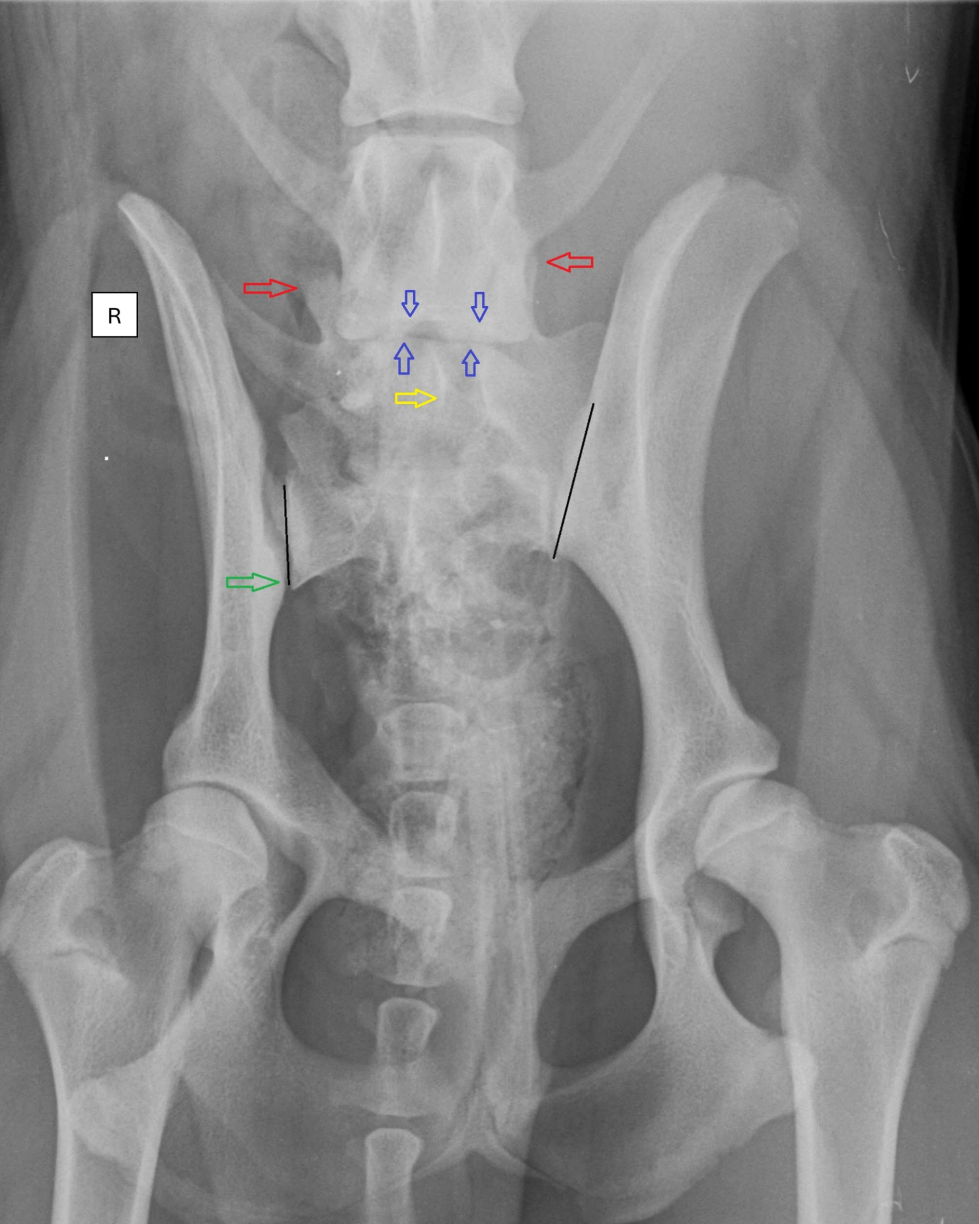
Ventrodorsal radiography with extended hips reveals an asymmetric lumbosacral transitional vertebra (LTV). The lumbosacral transitional vertebra (LTV) segment exhibits a sacral-like transverse process with a broad contact area on the left. The rotation of the LTV segment is evident on both the vertical and longitudinal axes, leaning towards the left. This is observed through the spinous process (yellow arrow), cranial articular facets (red arrows), and cranial and caudal endplates compared to the last normal lumbar vertebra (blue arrows). The pelvis demonstrates longitudinal axis rotation, noticeable in the wider left iliac wing and the asymmetric appearance of the obturator foramen. Additionally, a counterclockwise vertical rotation of the pelvis and sacrum is observed and a caudal displacement of the sacroiliac joint (green arrow). This displacement shortens the distance from the sacroiliac joint to the hip joint on the right side. Moreover, inadequate coverage of the left acetabulum leads to subluxation of the left hip joint. Axial malpositioning of a normal dog during the radiographic examination results in apparent rotation of the pelvis and the caudal lumbar spine in the same direction. However, if the direction or degree of the rotation, or both together, between the pelvis and lumbar spine are different, an inherent malposition should be considered [8]. The black lines demonstrate the measurement of the sacroiliac joint length. (The radiograph is for illustration purposes and not from the NKK database)

A shift in the position of the sacroiliac joint characterises the rotation of the pelvis vertically (PelvVert). In contrast, pelvis rotation about the longitudinal axis (PelvLong) is characterised by one wing of the ilium appearing wider than the contralateral side and most often, the ipsilateral obturator foramen appears smaller than the contralateral (Fig. 2). Hence, symmetry of the obturator foramina and equal width of the iliac wings were considered no pelvic rotation.

Rotation of the LTV segment about the vertical (LTVVert) and long axis (LTVLong) was evaluated based on the position of the transverse processes, spinous process, articular facets, and endplates of the last true lumbar vertebra (Fig. 2) [3, 7, 8].

### Measurements of anatomical landmarks for the positioning of the sacroiliac joints

A subsample of 743 dogs from the 14 breeds was selected (Fig. 1; Table 2), representing symmetrical and asymmetrical LTV types, to closely evaluate sacroiliac joint positioning and its relationship to LTV morphology. The sample size calculation was constrained by the smaller group size of dogs with asymmetrical LTV (*n* = 551).

**Table 2:** Information on the number of dogs within each breed in the subpopulation, including LTV morphology

Dog Breeds	mLTV	Counts	% of total
Brittany	Sym	40	5.4
	Asym	54	7.3
Bernese Mountain Dog	Sym	21	2.9
	Asym	15	2.0
Boxer	Sym	45	6.1
	Asym	7	1.0
Rough Collie	Sym	32	4.4
	Asym	12	1.6
Danish-Swedish Farm Dog	Sym	17	2.3
	Asym	27	3.7
English Setter	Sym	42	5.7
	Asym	37	5.0
Eurasier	Sym	18	2.4
	Asym	23	3.1
Gordon Setter	Sym	16	2.2
	Asym	23	3.1
Irish Setter	Sym	21	2.9
	Asym	26	3.5
Norwegian Elkhound Grey	Sym	20	2.7
	Asym	9	1.2
Portuguese Water Dog	Sym	19	2.6
	Asym	29	3.9
Rhodesian Ridgeback	Sym	18	2.4
	Asym	18	2.4
German Shepherd Dog	Sym	58	7.9
	Asym	53	7.2
Norwegian Elkhound Black	Sym	4	0.5
	Asym	39	5.2

The number of dogs within each breed and the lumbosacral transitional vertebra morphology (mLTV), where lumbosacral transitional vertebra (LTV) was classified into 3 classes [6] and then arranged in symmetrical (Sym) and asymmetrical (Asym) groups

To achieve a 95% confidence level, ensuring the true value falls within a margin of ± 5%, a minimum sample of 227 dogs with asymmetrical LTV was required. However, we ultimately sampled 372 dogs with asymmetrical LTV, maintaining a 95% confidence level with a population proportion of 67%. This larger sample size resulted in a narrower margin of error of ± 2.75%.

**Fig. 3:**
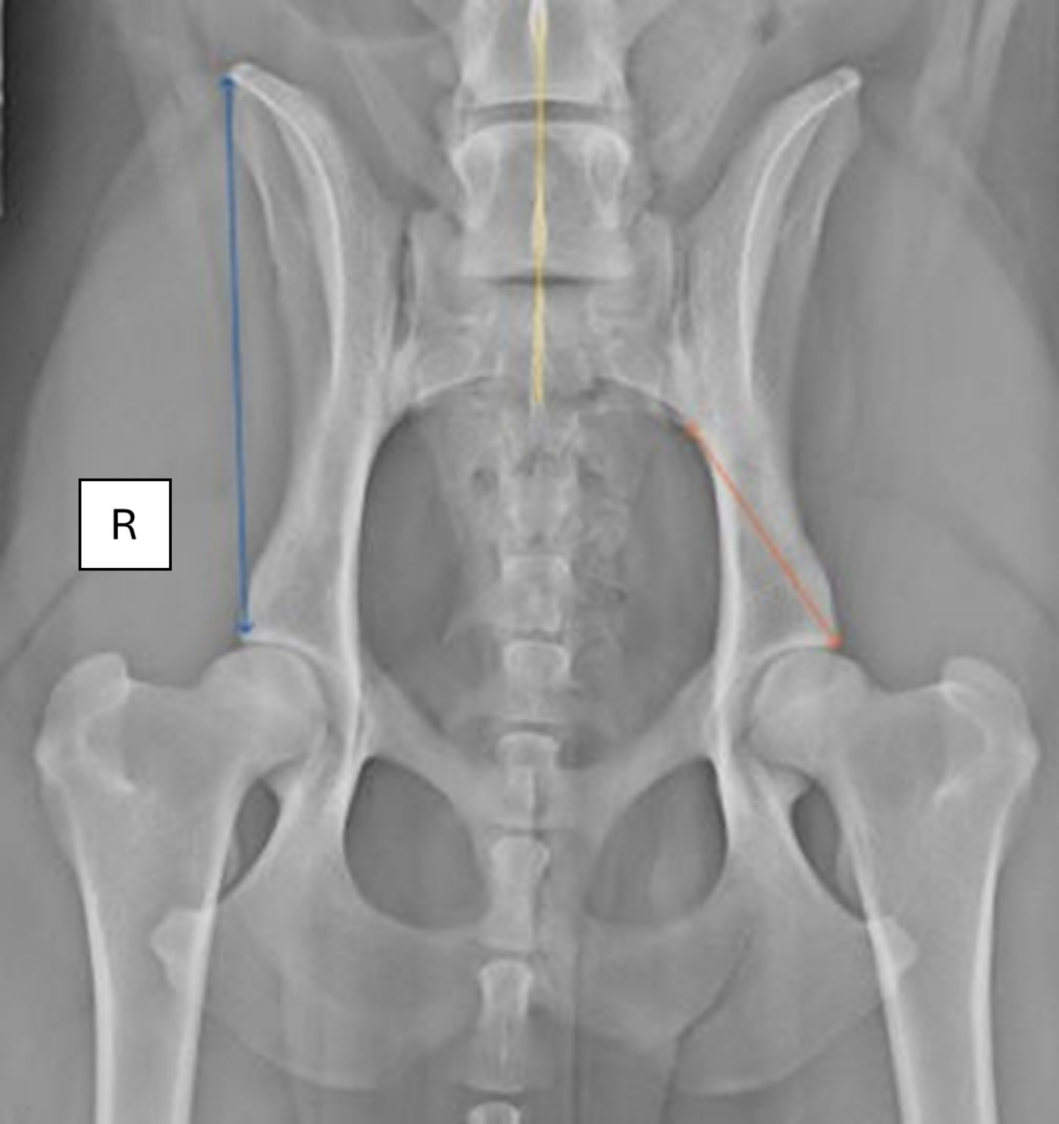
Ventrodorsal radiography with extended hips with normal anatomy position. Both transverse processes of the last lumbar vertebra are identically formed, oriented in craniolateral direction and have no contact with the pelvis. The length and position of the sacroiliac joints are identical. The yellow line represents the longitudinal axis. The blue double-ended arrow demonstrates the measurement of the ilium length. The double-ended orange arrow symbolises the measure of the distance from the sacroiliac joint to the hip joint. (The radiograph is for illustration purposes and not from the NKK database)

Measurements were performed bilaterally to quantify specific anatomical structures of the pelvis at defined anatomical points. The measured parameters included: (A) the length from the cranial tip of the ilium to the craniolateral acetabular rim on the left (IliumL) and right (IliumR) sides; (B) the length of the sacroiliac joint, i.e. including any parts of the LTV segment on the left (L-SJL) and right (L-SJR) side and (C) the length from the caudal end of the sacroiliac joint to the craniolateral acetabular rim on the left (Dsj-hjL) and right (Dsj-hjR) side (referred to as the distance from the sacroiliac joint to the hip joint in the text) (Figs. 2 and 3).

### Statistical analysis

Categorical data (LTV morphology, CHD grade, rotation of the LTV and pelvis in the vertical and long axis (i.e., no rotation, left or right rotation) are reported as frequencies (percentages). Continuous data (length of IliumL, IliumR, the L – SJL, L - SJR and the Dsj - hjL, Dsj - hjR) were evaluated for central tendencies, skewness, kurtosis and normal distribution.

For the statistical analyses, the LTV types were classified into two groups based on their morphology: (A) symmetrical (LTV types 1 and 2) and (B) asymmetrical (LTV type 3). In addition, the official FCI CHD grades were reclassified into four categories, where CHD grades A and B were grouped as free. CHD grades C, D, and E remained in the original categories. CHD grades were compared bilaterally, and each dog was classified as symmetrical (both sides were equal) or asymmetrical. Furthermore, the two sides were compared to determine the side with the worst CHD grade.

A bilateral multiple linear regression was used to test if the length of the left and right sacroiliac joint, length of the left and the right ilium, and morphology of the LTV were associated with the distance from the left and right sacroiliac joint to the respective left and right hip joint with the following formulas:

Dsj-hjL = β0 + (β1*LSJL) +( β2*IliumL) +( β3*IliumR) + (β4*mLTV) + ε, and.

Dsj-hjR = β0 + (β1*LSJR) + (β2*IliumL) + (β3*IliumR) + (β4*mLTV)+ ε.

The overall regression model for the left and right sides was statistically significant [(adjusted R^2^ = 0.376, F(2223) = 223, *P* < 0.001) and (adjusted R^2^ = 0.374, F(2227) = 227, *P* < 0.001)] for the left and right sides, respectively. The bilateral length of the sacroiliac joint was log transferred for a normal distribution, and there were no interactions between the predictors. There were no collinearity issues based on the variance inflation factor (VIF) and no influential outliers based on Cook’s distance, and the reference value was symmetrical LTV morphology.

We assessed pelvic rotation in the vertical axis using a multinomial logistic regression model: PelvVert = ~ 1 + mLTV + LTVLong + (mLTV*LTVLong).

The reference values were: no rotation for the pelvis in the vertical axis, symmetrical for LTV morphology, and no rotation for LTV in the long axis. The model was statistical significant (X²(10) = 882, *P* < 0.001), with a pseudo-R²_McF_ of 0.428.

The relationship between pelvic rotation in the long axis and LTV morphology, as well as LTV rotation in the long axis, was examined using a multinomial logistic regression model: PelvLong = ~ 1 + mLTV + LTVLong + (mLTV * LTVLong).

The model was statistically significant (X²(10) = 991, *P* < 0.001 and pseudo-R²_McF_ 0.460). The reference values were no rotation for pelvic, symmetrical for LTV morphology, and no rotation of the LTV on the long axis.

Furthermore, we examined the probability of developing asymmetrical CHD grades (assCHD) with the following binominal logistic regression equation:

Logit assCHD = β0 + (β1 * mLTV) + (β2 * LTVVert) + (β3 * LTVLong) + (β4 * PelvLong) + (β5 * PelvVert).

The model was statistically significant (X²(2) = 322, *P* < 0.001), with a pseudo-R^2^_McF_ of 16.60% and an area under the curve (AUC) of 87%. The reference values were symmetrical CHD grade, symmetrical LTV morphology, vertical LTV rotation: no rotation, and pelvic rotation over the long axis, which is no rotation.

The Chi-square test was used to identify any association between the hip joint with the worst grade and the pelvis rotation direction over the long axis.

All P-values less than 0.05 were rendered significant, odds ratios (OR) were calculated with 95% confidence intervals (Cl), and all data were analysed using commercial software (jamovi.org, version 2.3.18.0) [15].

## Results

A total of 13,950 VD radiographs were evaluated among 14 dog breeds, where 2579 (18.5%) dogs had LTVs. The majority, 2028 (78.6%), had symmetrical LTVs, whereas 551 (21.4%) had asymmetrical LTVs (Table 3).

**Table 3:** Information on the distribution of LTV morphology among 14 dog breeds

Dog Breeds	mLTV	Counts	% of total
Brittany	Sym	261	10.1
	Asym	82	3.2
Bernese Mountain Dog	Sym	75	2.9
	Asym	18	0.7
Boxer	Sym	137	5.3
	Asym	11	0.4
Rough Collie	Sym	172	6.7
	Asym	17	0.7
Danish-Swedish Farm Dog	Sym	112	4.3
	Asym	37	1.4
English Setter	Sym	129	5.0
	Asym	49	1.9
Eurasier	Sym	140	5.4
	Asym	34	1.3
Gordon Setter	Sym	78	3.0
	Asym	35	1.4
Irish Setter	Sym	90	3.5
	Asym	47	1.8
Norwegian Elkhound Grey	Sym	220	8.5
	Asym	20	0.8
Portuguese Water Dog	Sym	106	4.1
	Asym	40	1.6
Rhodesian Ridgeback	Sym	191	7.4
	Asym	32	1.2
German Shepherd Dog	Sym	167	6.5
	Asym	76	2.9
Norwegian Elkhound Black	Sym	150	5.8
	Asym	53	2.1

The number of dogs within each breed and the lumbosacral transitional vertebra morphology (mLTV), where lumbosacral transitional vertebra (LTV) was classified into 3 classes [6] and then arranged in symmetrical (Sym) and asymmetrical (Asym) groups

Out of the 551 asymmetrical LTVs, 261 dogs (47.36%) did not exhibit longitudinal axis rotation, while 158 dogs (28.31%) showed a rotation towards the right, and 132 dogs (23.97%) exhibited a rotation towards the left about the longitudinal axis. Table 4 provides further details related to the vertical and longitudinal axis rotation of the LTV and pelvis, along with the morphology of the LTV segment.

**Table 4:** The rotation of the LTV segment and pelvis and their relationship to LTV morphology

LTVLong	mLTV	Counts	% of total	LTVVert	mLTV	Counts	% of total
No	Sym	1962	76.1	No	Sym	1984	76.9
	Asym	261	10.1		Asym	251	9.7
Right	Sym	35	1.4	Right	Sym	17	0.7
	Asym	158	6.1		Asym	169	6.6
Left	Sym	31	1.2	Left	Sym	27	1.0
	Asym	132	5.1		Asym	131	5.1
PelvLong				PelvVert			
No	Sym	1975	76.6	No	Sym	1993	77.2
	Asym	325	12.6		Asym	323	12.6
Right	Sym	17	0.7	Right	Sym	22	0.9
	Asym	119	4.6		Asym	120	4.7
Left	Sym	36	1.4	Left	Sym	14	0.5
	Asym	107	4.1		asym	107	4.2

The distribution of the vertical and long axis rotation of the lumbosacral transitional vertebra (LTV) and the pelvis, among the LTV morphology, where LTV was classified into 3 classes [6] and then arranged in symmetrical and asymmetrical groups. LTVLong, rotation of the LTV segment about the longitudinal axis, no, right and left rotation. mLTV, the morphology of the LTV segment, and its symmetrical or asymmetrical appearance. LTVVert, rotation of the LTV segment in the vertical axis. PelvLong, rotation of the pelvis about the longitudinal axis. PelvVert, rotation of the pelvis about the vertical axis

A total of 743 dogs (28.8%) had measurements of specified pelvic landmarks, of which 371 (49.9%) exhibited symmetrical LTV and 372 (51.1%) asymmetrical LTV, respectively.

Among the 2579 dogs with an LTV, 320 (12.4%) had an asymmetric CHD grade; 193 (60.3%) of these dogs had symmetrical, and 127 (39.7%) had asymmetrical LTV. Hence, there were 1835 (71.2%) dogs with symmetrical CHD and symmetrical LTV, while there were 424 (16.4%) with asymmetrical LTV.

Further details regarding symmetrical CHD and LTV are provided in Table 5. Among the 320 dogs with an asymmetric CHD grade, 49 (15.31%) exhibited asymmetric LTV and pelvic rotation about the longitudinal axis towards the right. Conversely, 55 dogs (17.18%) demonstrated asymmetrical LTV with the pelvis rotated towards the left about the longitudinal axis. At the same time, 27 (7.21%) did not exhibit any longitudinal axis rotation. Further details related to rotational changes of the LTV, pelvis and asymmetric CHD grades are provided in Table 5.

**Table 5:** The relationship between pelvic longitudinal axis rotation, LTV morphology, and symmetrical or asymmetrical hip grades

PelvLong	mLTV	CHD L – CHD *R*	Counts	% of total
No	Sym	Sym	1823	70.7
	Asym		302	11.7
	Sym	Asym	152	5.9
	Asym		23	0.9
Right	Sym	Sym	10	0.4
	Asym		70	2.7
	Sym	Asym	7	0.3
	Asym		49	1.9
Left	Sym	Sym	2	0.1
	Asym		52	2.0
	Sym	Asym	34	1.3
	Asym		55	2.1

The table illustrates the number of dogs with pelvic rotation about the longitudinal axis, with symmetrical and asymmetrical lumbosacral transitional vertebra morphology, against symmetrical or asymmetrical canine hip dysplasia (CHD) grades. CHD grade was according to FCI [14]. For statistical purposes, CHD grades A and B were united into one group, “free”. Lumbosacral transitional vertebra (LTV) was classified into 3 classes [6] and then grouped based on morphology; LTV type 1 and LTV type 2 are symmetrical, while LTV type 3 is asymmetrical. PelvLong, rotation of the pelvis about the longitudinal axis, mLTV, the morphology of the LTV segment, and its symmetrical or asymmetrical appearance, CHD L, CHD R, left and right canine hip dysplasia grade, Sym means equal (symmetrical) CHD grade, Asym means (asymmetrical) unequal CHD grade

With an asymmetrical LTV, the shorter sacroiliac joint was positioned further caudally on the pelvis, reducing the distance between the sacroiliac joint and the hip joint compared to the opposite, longer sacroiliac joint. The length from the tip of the ilium to the acetabulum did not differ between the left and right sides, indicating equal positioning of the acetabulum bilaterally. The statistical results were as follows for the left and right sides, respectively: (left: β = 10.94, *P* < 0.001; right: β = 12.86, *P* < 0.001) and for the morphology of the LTV segment (left: β = -3.79, *P* < 0.001; right: β = -3.59, *P* < 0.001).

Rotation of the asymmetric LTV about the longitudinal axis was associated with a pelvic rotation contralateral to the LTV segment vertically right [OR = 9.36, *P* < 0.001, (95% CI [6.35–13.79])], left [OR = 6.27, *P* < 0.001, (95% CI [3.93–9.97])], indicating that when an asymmetrical LTV rotates about the long axis, the pelvis tends to rotate in the opposite direction vertically.

Rotation of the asymmetric LTV about the longitudinal axis was associated with rotation of the pelvis about the longitudinal axis, causing an elevation of the pelvis on the right side [OR = 4.94, *P* < 0.001, (95% CI [3.10–7.86])], left side [OR = 5.46, *P* < 0.001, (95% CI [3.52–8.56])].

An asymmetrical CHD grade was associated with pelvic rotation about the longitudinal axis (right rotation) [OR = 8.50, *P* < 0.001, (95% CI [5.84–12.36])]; (left rotation) [OR = 19.94, *P* < 0.001, (95% CI [13.79–29.07])]. Furthermore, we found an association between the rotational direction of the pelvis about the longitudinal axis and the side with the worst CHD grade; when the worst CHD grade was on the left, this was statistically significantly associated with elevation of the pelvis on the left side [X²(2, *n* = 2579) = 509, *P* < 0.001], while for the contralateral hip, when the worst CHD grade was on the right side, there was an associated elevation of the pelvis on the right side [X²(2, *n* = 2579) = 311, *P* < 0.001].

## Discussion

This study supports our hypothesis that a caudally positioned shorter sacroiliac joint with an asymmetrical LTV caused a reduced distance between the sacroiliac joint and the hip joint; however, the length of the iliac wing and, hence, the positioning of the acetabulum in relation to the length of the iliac wing was not affected. This results in a reduced distance for the propulsive forces from the pelvic limb to the spine, which reduces the moment acting on the sacroiliac joint based on the equation “Moment = Force*Distance” and might explain the findings that there was no association between the presence of an asymmetric LTV and degenerative changes in the sacroiliac joint [13]. Nevertheless, vigorous exercises with normal sacroiliac anatomy are associated with degenerative changes in the sacroiliac joint [13, 16].

Our results, supported by previous indications, show that the sacrum and the pelvis rotate in the opposite direction to the asymmetric LTV segment about the longitudinal axis [3]. The occurrence of counter-rotation could be a compensatory response to mitigate the loss of normality. This phenomenon may be a way to straighten out the lower back and pelvis in the situation of a skewed skeleton [17–20]. The head-tail line could cause an extremely abnormal posture if this compensation did not occur. Nevertheless, this counter rotation might predispose to CES as the anatomical structures are “twisted” on each other [3, 10, 12].

Our results show that an asymmetrical LTV might cause pelvic rotation about the longitudinal axis and promote asymmetric CHD-grade development. The findings also support previous findings that the worst CHD grade is on the side where the pelvis is elevated, in the standing dog [8, 21]. Our study contradicts previous findings, in which the side with the worst hip is where the transverse process attaches to the ilium [7, 8]. This disparity might be explained by recognising that some symmetrical LTV might promote a less pronounced pelvis rotation in both the vertical and long axes. This aligns with previous publications [8, 9]. This might also explain the disparity in the results between the left and right sides regarding asymmetrical LTV and asymmetric CHD grades.

We could not identify an association between an asymmetric CHD grade and rotation of the pelvis vertically, which is reported in some other studies [7, 8], nor could we identify any association between LTV morphology and asymmetric CHD grade [8]. This could be because of different classification systems related to the grading of LTVs as well as the CHD grade; for instance, the Swiss CHD grade is more extensive than the FCI system [7, 8, 14, 22].

In this study, only VD projections of the pelvis were available. In cases of longitudinal axis misalignment during the radiographic examination of a normally positioned dog, it can lead to the appearance of rotation in the pelvis with the caudal lumbar spine in the same direction. However, a direction or degree of rotation difference between the pelvis and the lumbar spine suggests an inherent misalignment [8]. Meanwhile, radiographs taken in lateral recumbency could assist in distinguishing the cause of rotation [4]. For a more accurate identification of the underlying cause of misalignment, computed tomography imaging may be employed, but it is not feasible for mass screening purposes [4].

Furthermore, a disparity between symmetrical and asymmetrical CHD grades concerning longitudinal axis pelvic rotation suggests a potential association with asymmetrical CHD grade. While a plausible explanation is presented in the text, it is essential to approach the interpretation of this result with some caution.

Measuring the length of the pelvis in dogs of different sizes will naturally yield varying measurements. However, this variation will not affect the results if asymmetric LTV causes the different lengths of the sacroiliac joint. These differences will be evident regardless of the overall pelvic size of the dogs.

## Conclusions

The present study indicates that an asymmetrical LTV is associated with rotation of the LTV segment, which might promote pelvic rotation in the vertical and longitudinal axes. With an asymmetrical LTV rotation in the longitudinal axis, the pelvis rotates vertically in the opposite direction. Furthermore, our findings indicate that asymmetric LTV potentially induces rotation within the same longitudinal axis orientation of the pelvis, causing a unilateral elevation of the pelvis, which contributes to the promotion of an uneven CHD grade. Finally, an asymmetrical LTV influences the sacroiliac joint contact area, where the side with the most diminutive sacroiliac joint seems to be protected by moving the assembly caudally, reducing the length from the sacroiliac joint to the hip joint.

## Data Availability

The datasets used and analysed during the current study are available from the corresponding author on reasonable request.

## Declarations

### Prior publication

Data have not been published previously.

## References

[1] Larsen JS. Lumbosacral Transitional vertebrae in the dog. Vet Radiol Ultrasound. 1977;18:76–9. 10.1111/j.1740-8261.2005.00103.x 16429983

[2] Winkler W, Loeffler K. Lumbosacral transitional vertebrae in the dog. Berl Munch Tierarztl Wochenschr. 1986;99:343–6. 3790061

[3] Damur-Djuric N, Steffen F, Hässig M, Morgan JP, Flückiger MA. Lumbosacral transitional vertebrae in dogs: classification, prevalence, and association with sacroiliac morphology. Vet Radiol Ultrasound. 2006;47:32–8. 16429982 10.1111/j.1740-8261.2005.00102.x

[4] Lappalainen AK, Salomaa R, Junnila J, Snellman M, Laitinen-Vapaavuori O. Alternative classification and screening protocol for transitional lumbosacral vertebra in German shepherd dogs. Acta Vet Scand. 2012;54:27. 22549019 10.1186/1751-0147-54-27 PMC3403972

[5] Morgan JP. Congenital anomalies of the vertebral column of the dog: a study of the incidence and significance based on a radiographic and morphologic study. Vet Radiol. 1968;9:21–9.

[6] Flückiger M, Geissbühler U, Lang J. Lumbosakrale übergangswirbel: welche bedeutung haben sie für die gesundheit von betroffenen hunden? Schweiz Arch Tierheilkd. 2009;151:133–5. 19263383 10.1024/0036-7281.151.3.133

[7] Morgan JP. Transitional lumbosacral vertebral anomaly in the dog: a radiographic study. J Small Anim Pract. 1999;40:167–72. 10340246 10.1111/j.1748-5827.1999.tb03784.x

[8] Flückiger MA, Steffen F, Hässig M, Morgan JP. Asymmetrical lumbosacral transitional vertebrae in dogs may promote asymmetrical hip joint development. Vet Comp Orthop Traumatol. 2017;30:137–42. 28094414 10.3415/VCOT-16-05-0072

[9] Berg JA, Sævik BK, Lingaas F, Trangerud C. Lumbosacral transitional vertebra in 14 dog breeds in Norway: occurrence, risk factors and association with hip dysplasia. Vet J. 2024;303:106056. 38092176 10.1016/j.tvjl.2023.106056

[10] Morgan JP, Bahr A, Franti CE, Bailey CS. Lumbosacral transitional vertebrae as a predisposing cause of cauda equina syndrome in German shepherd dogs: 161 cases (1987–1990). J Am Vet Med Assoc. 1993;202:1877–82. 8320160

[11] Flückiger MA, Damur-Djuric N, Hassig M, Morgan JP, Steffen F. A lumbosacral transitional vertebra in the dog predisposes to cauda equina syndrome. Vet Radiol Ultrasound. 2006;47:39–44. 16429983 10.1111/j.1740-8261.2005.00103.x

[12] Steffen F, Berger M, Morgan JP. Asymmetrical, transitional, lumbosacral vertebral segments in six dogs: a characteristic spinal syndrome. J Am Anim Hosp Assoc. 2004;40:338–44. 15238565 10.5326/0400338

[13] Komsta R, Łojszczyk-Szczepaniak A, Dębiak P. Lumbosacral transitional vertebrae, canine hip dysplasia, and sacroiliac joint degenerative changes on ventrodorsal radiographs of the pelvis in police working German shepherd dogs. Top Companion Anim Med. 2015;30:10–5. 26041591 10.1053/j.tcam.2015.02.005

[14] Fédération Cynologique Internationale. http:www.fci.be/en/. Accessed Apr 7 2022.

[15] Jamovi - open. statistical software for the desktop and cloud. https://www.jamovi.org/. Accessed Oct 31 2022.

[16] Breit S, Knaus I, Künzel W. The gross and radiographic appearance of sacroiliac ankylosis capsularis ossea in the dog. Res Vet Sci. 2003;74:85–92. 12507570 10.1016/s0034-5288(02)00156-x

[17] Millner PA, Dickson RA. Idiopathic scoliosis: biomechanics and biology. Eur Spine J. 1996;5:362–73. 8988378 10.1007/BF00301963

[18] Marlow TJ, Brunson CY, Jackson S, Schabel SI. Tower vertebra: a new observation in sickle cell disease. Skeletal Radiol. 1998;27:195–8. 9592901 10.1007/s002560050364

[19] Shapiro F. Fractures of the femoral shaft in children: the overgrowth phenomenon. Acta Orthop Scand. 1981;52:649–55. 7331804 10.3109/17453678108992162

[20] de Reuver S, IJsseldijk LL, Homans JF, Willems DS, Veraa S, van Stralen M, et al. What a stranded whale with scoliosis can teach us about human idiopathic scoliosis. Sci Rep. 2021;11:7218. 33785866 10.1038/s41598-021-86709-x PMC8009909

[21] Morgan JP, Stephens M. Radiographic diagnosis and control of canine hip dysplasia. ISUP. 1985;19:144–7.

[22] Flückiger M. Die standardisierte beurteilung Von röntgenbildern Von Hunden auf hüftgelenksdysplasie. Kleintierpraxis. 1993;38:693–702.

